# The impact of human-AI co-creation on perceived creativity in generative AI art: a serial mediation mechanism of psychological ownership, perceived agency, and creative flow

**DOI:** 10.3389/fpsyg.2026.1986050

**Published:** 2026-10-09

**Authors:** Chenyang Wang, Miao Chen, Wen Yang

**Affiliations:** 1 School of New Media Art, Xi’an Polytechnic University, Xi’an, Shaanxi, China; 2 School of Country and Region Studies, Northwest University, Xi’an, Shaanxi, China

**Keywords:** creative flow, digital art, generative artificial intelligence, human-AI co-creation, perceived agency, perceived creativity, psychological ownership

## Abstract

**Introduction:**

Generative AI is shifting digital art from one-step generation toward interactive human-AI co-creation. Across two between-subject experiments (*N* = 600), we examined how creation mode and prespecified AI-involvement workflows related to perceived creativity.

**Methods:**

In Study 1, participants completed digital art tasks under human-only creation, AI-only generation, or human-AI co-creation conditions. In Study 2, three workflows differing in AI involvement (low, moderate, high) were evaluated. Psychological ownership, perceived agency, creative flow, and perceived creativity were measured.

**Results:**

In Study 1, human-AI co-creation produced higher perceived-creativity ratings than AI-only generation, with indirect effects consistent with a serial pathway through psychological ownership, perceived agency, and creative flow. In Study 2, the moderate-involvement workflow yielded higher perceived-creativity ratings and psychological states than the low- and high-involvement workflows.

**Discussion:**

Perceived creativity in generative-AI art depends on how users experience contribution, control, and engagement during creation. Within the workflows tested, balancing generative assistance with consequential user choice fosters the most favorable subjective creativity evaluations.

## 1 Introduction

Generative artificial intelligence is transitioning from a mere automated generation tool to a human-AI co-creation system, thereby reshaping the relationships between “humans,” “tools,” and “artworks” in digital creation. Traditional digital art tools primarily served supportive functions, with users completing works through drawing, editing, layout, or post-processing; in contrast, within generative AI contexts, users continuously participate in the image generation process through prompt input, stylistic selection, multi-round modifications, localized adjustments, and result curation. In other words, generative AI does not simply replace human visual output; it increasingly becomes a collaborative system that participates in ideation, generation, feedback, and optimization. Computational creativity research has long indicated that AI can support creative generation by combining, exploring, and transforming existing representational spaces (Boden, 1998). Furthermore, the value of creativity support tools lies not only in efficiency gains but also in expanding users’ capacity to explore ideas, compare schemes, and express intentions (Shneiderman, 2007). In human-AI co-creative research, the role of AI systems is shifting from “tool” to “creative partner,” where interaction patterns, feedback mechanisms, and controllability influence users’ understanding and evaluation of the collaborative process (Lubart, 2005; Kantosalo and Toivonen, 2016; Rezwana and Maher, 2023). Recently, the proliferation of text-to-image models and multimodal generative systems has accelerated the integration of generative AI into digital art, visual communication, design education, and platform content production (Epstein et al., 2023). In this evolving landscape, prompt engineering and iterative image prompting have fundamentally transformed user interaction from passive browsing into an exploratory creative dialogue (Oppenlaender, 2022; Anantrasirichai and Bull, 2022). Consequently, evaluating generative outputs increasingly requires examining both the algorithmic capabilities and the collaborative synergy between human intent and machine generation (Franceschelli and Musolesi, 2024). Recent work in design innovation and technology ecosystems likewise emphasizes that AI can augment human ideation and decision-making when it is embedded in participatory workflows (Agboola, 2024; Baby et al., 2026). Thus, the core psychological question has evolved beyond “can AI create art?” to “how do users perceive their creative contributions and evaluate joint outputs when collaborating with AI?”

Existing research on AI art and generative content has primarily focused on aesthetic evaluation discrepancies between AI-generated and human-created works, AI label effects, algorithmic bias, authenticity judgments, and user acceptance. Relevant studies suggest that evaluations of AI art depend not only on visual quality but also on authorship, creative intent, source disclosure, and social cognitive biases (Moffat and Kelly, 2006; Chamberlain et al., 2018; Hong and Curran, 2019; Bellaiche et al., 2023; Horton et al., 2023). For instance, when works are labeled as AI-generated, users may downgrade evaluations of their artistic value, originality, or emotional depth; yet, under certain conditions, AI art may receive positive evaluations due to novelty, visual complexity, or technical imagination. While these studies provide a foundation for understanding the aesthetic reception and social cognition of AI art, notable gaps remain. First, many studies position users as “observers” or “evaluators,” focusing on their responses to completed AI works, with limited attention to the psychological changes occurring when users participate as co-creators in the prompt, selection, modification, and generation stages. Second, existing literature often treats perceived creativity as a final evaluation outcome without sufficiently explaining its formation during the creative process. Creativity is typically understood as the confluence of novelty and appropriateness or value (Amabile, 1982; Runco and Jaeger, 2012); however, in human-AI co-creative contexts, users’ judgments of creativity may stem not only from the output itself but also from self-related experiences during creation. Psychological ownership theory suggests that an individual’s control, investment, and self-extension foster a sense of “mine-ness” regarding an object (Pierce et al., 2001, 2003; Peck and Shu, 2009). In digital and technology-mediated environments, this psychological sense of ownership is primarily triggered when users perceive sufficient behavioral investment and individual control over the system’s interactive features (Jussila et al., 2015; Kirk et al., 2015). Perceived agency research emphasizes that whether an individual perceives themselves as the initiator of an action and its result influences their sense of control, responsibility attribution, and self-relevance (Synofzik et al., 2008; Moore, 2016; Haggard, 2017). When interacting with autonomous systems, delegating excessive decision-making to algorithms can degrade the user’s subjective sense of agency, whereas controllable and feedback-rich interfaces help maintain perceived shared agency (Berberian et al., 2012; Amershi et al., 2019). Flow theory posits that when individuals maintain high engagement through clear goals, immediate feedback, and moderate challenges, they are more likely to achieve focused immersion and process enjoyment (Csikszentmihalyi, 1990; Hoffman and Novak, 1996; Nakamura and Csikszentmihalyi, 2002; Novak et al., 2000). However, existing research has not fully integrated psychological ownership, perceived agency, and creative flow to explain perceived creativity in human-AI co-creation, a deficiency that forms the theoretical entry point for this study. Recent GenAI-assisted composition and design-education studies connect ownership, flow, and creativity, but do not test the proposed serial process in generative visual-art co-creation (Tong, 2026; Tsakalerou et al., 2026).

To address these gaps, this study proposes a serial mediation model to explain the impact of human-AI co-creation on perceived creativity. Specifically, human-AI co-creation may first enhance the user’s psychological ownership of the work, making them feel more strongly that the final output reflects their intentions, choices, and investment; this psychological ownership further promotes perceived agency during creation—the user’s belief that they can influence the direction and outcome of the work; high perceived agency subsequently facilitates creative flow, enabling the user to experience greater focus, immersion, and process enjoyment, ultimately enhancing the perception of the work’s creativity. Such flow experiences require an optimal balance between perceived task challenges and individual operational capabilities, which directly fosters heightened cognitive absorption and creative intrinsic motivation (Jackson and Marsh, 1996; Peifer et al., 2014). From an interaction perspective, whether an AI system provides interpretable feedback, controllable operations, and continuous interaction influences whether the user perceives it as a passive tool, an autonomous system, or a collaborative partner (Amershi et al., 2019; Rezwana and Maher, 2023). From a creativity evaluation perspective, user judgments may concurrently include a synthesis of the work’s novelty, appropriateness, personal investment, and the experience of the creative process (Amabile, 1982; Runco and Jaeger, 2012). To test this mechanism, this study conducts two experiments: Study 1 compares the impacts of human-only creation, AI-only generation, and human-AI co-creation on perceived creativity and relevant psychological variables; Study 2 further examines the impact of varying levels of AI involvement to test whether moderate AI involvement is more conducive to positive creative evaluations than low or high AI involvement. Based on this, we propose the following hypotheses:

Against this background, the study makes three bounded advances. First, it moves beyond comparisons of human- and AI-labeled outputs by examining how participation in the creative workflow shapes perceived creativity. Second, it connects psychological ownership, perceived agency, and creative flow in a single theoretically ordered indirect-effect model. Third, it distinguishes creation mode from the specific distribution of assistance and user control, allowing Study 2 to test whether the moderate workflow differs from both lower- and higher-involvement workflows. These advances concern subjective creativity judgments within the tested digital-art task and do not assume equivalence with independently assessed creative performance.

*H1*: Human-AI co-creation is associated with higher perceived creativity compared to AI-only generation.

*H2*: Human-AI co-creation is positively related to psychological ownership.

*H3*: Psychological ownership positively predicts perceived agency.

*H4*: Perceived agency positively predicts creative flow.

*H5*: Creative flow positively predicts perceived creativity.

*H6*: Psychological ownership, perceived agency, and creative flow serially mediate the relationship between human-AI co-creation and perceived creativity.

*H7*: Moderate AI involvement yields higher perceived creativity evaluations than either low or high AI involvement.

## 2 Materials and methods

### 2.1 Overview of research design

This study used two related between-subjects experiments to examine perceived creativity in generative-AI art. Study 1 manipulated creation mode by comparing human-only creation, AI-only generation, and human-AI co-creation. Study 2 focused on three prespecified co-creative workflows that differed in AI support and user interaction permissions. Together, the studies tested whether participation in co-creation was associated with perceived creativity and whether this relationship was consistent with a serial process involving psychological ownership, perceived agency, and creative flow.

Study 1 used a one-factor, three-group between-subjects design in which creation mode defined the division of creative activity between the participant and the system. Participants in the human-only condition developed and submitted an artwork concept without AI generation. Participants in the AI-only condition viewed system-generated candidates and selected a final image without editing prompts or regenerating outputs. Participants in the human-AI co-creation condition could enter prompts, select styles, generate multiple outputs, and curate the final image. Study 2 used a one-factor, three-group between-subjects design. Its low-, moderate-, and high-involvement conditions represented different bundles of AI support, prompt control, regeneration opportunities, and final-selection authority. Table 1 summarizes the user role, AI role, and operational permissions in each condition.

**Table 1:** Experimental conditions and manipulation methods.

Study	Condition	User role	AI role	Primary manipulation method
Study 1	Human-only creation	Independently conceive and complete the artwork proposal	No participation in generation	User is the sole creative agent
	AI-only generation	View or select from system-provided results	Automatically generate the primary artwork	User does not participate in the generation process
	Human-AI co-creation	Input prompts, select styles, execute multi-round modifications	Generate and optimize the artwork based on user feedback	User and AI collaboratively form the artwork
Study 2	Low AI involvement	Lead creative decisions	Provide only limited suggestions	Weak AI support, high user control
	Moderate AI involvement	Engage in multi-round interaction with AI while retaining key choices	Provide generation, feedback, and variations	Highest degree of collaborative synergy
	High AI involvement	Primarily select or confirm results	Complete the majority of generative tasks	High degree of AI dominance

Both experiments assessed perceived creativity, psychological ownership, perceived agency, creative flow, aesthetic evaluation, and emotional resonance. In this manuscript, creation mode denotes the categorical allocation of creative activity between the participant and AI in Study 1. AI-involvement condition denotes the three operational workflows tested in Study 2; it is not treated as a direct continuous measure of automation. Perceived creativity denotes participants’ post-task judgments of their own final work, whereas external creativity ratings denote evaluations made by condition-blind raters. Study 1 tested differences among creation modes and the proposed indirect-effect model. Study 2 tested whether the moderate workflow produced more favorable evaluations than the low- and high-involvement workflows. Figure 1 summarizes the experimental grouping and theoretical model.

**Figure 1:**
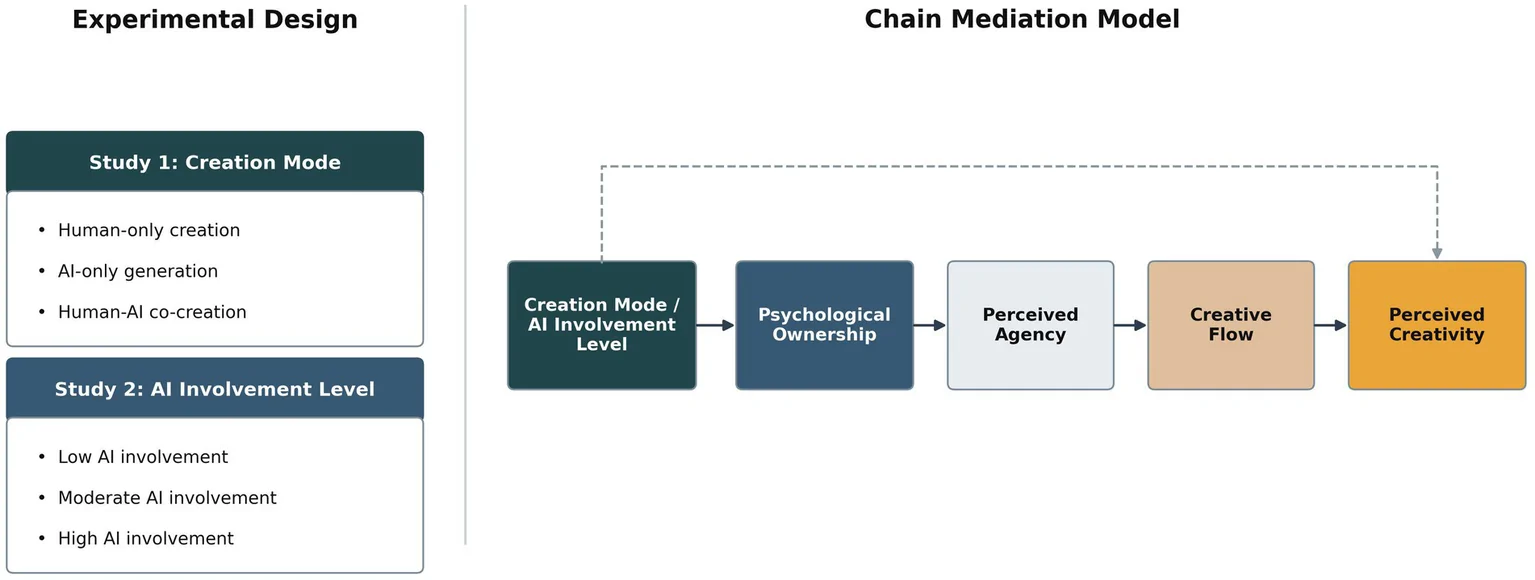
Overview of the theoretical model and experimental design.

### 2.2 Participants

Study 1 included 300 valid participants, with an average age of 24.39 years (SD = 3.99). The sample comprised 150 females (50.0%), 144 males (48.0%), and 6 individuals who identified as other or preferred not to disclose (2.0%). Regarding educational background, 173 were undergraduate students (57.7%), 61 were master’s students (20.3%), 57 were junior college students (19.0%), and 9 were doctoral students (3.0%). Participants reported a mean generative AI usage experience of 2.33 (SD = 1.67) and a mean art/design experience of 2.11 (SD = 1.58).

Study 2 similarly included 300 valid participants, with an average age of 24.28 years (SD = 3.99). The sample consisted of 155 males (51.7%), 136 females (45.3%), and 9 individuals who identified as other or preferred not to disclose (3.0%). In terms of education, 181 were undergraduate students (60.3%), 62 were master’s students (20.7%), 39 were junior college students (13.0%), and 18 were doctoral students (6.0%). The mean generative AI usage experience was 2.47 (SD = 1.72), and the mean art/design experience was 1.90 (SD = 1.42).

Participants were recruited in China via Credamo, a professional academic survey platform with quality-control mechanisms. The experimental system and all measurement scales were administered in Simplified Chinese. Participants took approximately 10 to 12 min to complete the creation task and psychological questionnaire. Each participant who passed the attention checks and met the inclusion criteria received RMB 10 (approximately USD 1.40).

The target sample size was determined by an *a priori* power analysis for a one-factor, three-group analysis of variance. Assuming a medium effect size (*f* = 0.25), alpha = 0.05, and power of 0.95, the analysis indicated a minimum total sample of approximately 252 participants per study. To accommodate exclusions and provide sufficient precision for bootstrap indirect-effect estimates and covariate-adjusted models, the target valid sample was increased to 300 participants per study, with 100 planned participants per condition.

All participants read the research instructions and signed an electronic informed consent form before the experiment commenced. Participants were informed that the study focused on digital art creation experiences and artwork evaluation; participation was entirely voluntary, and they reserved the right to withdraw at any stage. All data were collected and analyzed anonymously, strictly for academic research purposes. This study was conducted in compliance with the institutional ethical guidelines for anonymous online behavioral and questionnaire-based research. The demographic information of the participants for both experiments is detailed in Table 2.

**Table 2:** Demographic information of participants.

Study	Sample size	Age (M ± SD)	Gender	Education level	Generative AI usage experience (M ± SD)	Art/design experience (M ± SD)
Study 1	300	24.39 ± 3.99	Female 150 (50.0%); Male 144 (48.0%); Other/Not Disclosed 6 (2.0%)	Undergraduate 173 (57.7%); Master’s 61 (20.3%); Junior College 57 (19.0%); Doctoral 9 (3.0%)	2.33 ± 1.67	2.11 ± 1.58
Study 2	300	24.28 ± 3.99	Male 155 (51.7%); Female 136 (45.3%); Other/Not Disclosed 9 (3.0%)	Undergraduate 181 (60.3%); Master’s 62 (20.7%); Junior College 39 (13.0%); Doctoral 18 (6.0%)	2.47 ± 1.72	1.90 ± 1.42

### 2.3 Experimental materials and stimuli

This study employed digital art creation as the experimental task. To minimize the confounding effects of thematic variance on perceived creativity, aesthetic evaluation, and emotional resonance, all participants were required to complete an artwork—through generation, selection, or ideation—centered on a unified theme: “The Symbiosis of Future Cities and Nature.” The task instructions presented to the participants were as follows: “Please complete a digital art proposal centered on the theme ‘The Symbiosis of Future Cities and Nature.’ The work should embody a clear visual theme and demonstrate novelty and expressiveness to the greatest extent possible.” Prior to the task, all participants viewed identical theme descriptions, grading criteria prompts, and two baseline example artworks. The example artworks served exclusively to illustrate fundamental visual quality requirements; to mitigate priming and anchoring effects, they did not provide directly replicable prompts, compositional layouts, or style templates.

The generative AI image generation service was built upon the Stable Diffusion XL 1.0 model (API calls were executed in March 2026). All AI generation conditions were restricted to the unified theme and utilized identical backend generation parameters: an image resolution of 1,024 × 1,024 pixels, 30 sampling steps, a Classifier-Free Guidance (CFG) scale of 7.5, and the DPM++ 2 M Karras sampler. Differences between conditions were strictly limited to user interaction permissions, specifically whether the user could input or modify prompts, select styles, execute multi-round re-generations, and curate results. The prompt templates, style options, negative prompt configurations, and representative generative outputs used in the experiment are provided in the Supplementary material.

Both experiments were administered via a unified web-based experimental system. The experimental materials comprised task instructions, the generative AI image generation/display interface, the artwork presentation interface, operational guidelines, psychological measurement questionnaires, and a behavioral logging module. To ensure comparability across conditions, the task theme, basic page structure, questionnaire content, and the final artwork confirmation workflow were kept consistent across all groups. Variations across conditions were solely reflected in whether users were permitted to input/modify prompts, select styles, perform multi-round re-generations, or edit results, as well as the extent to which the final artwork was determined by the user versus the AI.

Study 1 differentiated three creation conditions by manipulating the creative agent and the mode of generative participation. In the human-only creation condition, AI generation, prompt-based generation, and re-generation permissions were disabled; participants primarily relied on the task theme to independently conceive their artwork and confirm the final proposal. In the AI-only generation condition, prompt editing, multi-round modification, and local adjustment functions were disabled; participants viewed candidate works automatically generated by the system based on the unified theme and selected one as their final piece. In the human-AI co-creation condition, functions for prompt input, style selection, re-generation, and result curation were fully enabled; participants could input or modify prompts, select visual styles, and confirm the final work from multiple rounds of generative outputs.

Study 2 further manipulated the level of AI involvement. Under the low AI involvement condition, the AI provided only limited thematic associations or compositional suggestions; it did not directly lead the generation of the final artwork, and the primary creative decisions were executed by the user. Under the moderate AI involvement condition, participants were permitted to input and modify prompts, select styles, and engage in multiple rounds of generation and result curation; the AI provided generative support, but the final decisions regarding prompts, stylistic direction, and artwork confirmation remained with the user. Under the high AI involvement condition, the system automatically generated the primary candidate works based on the task theme; participants could not freely edit prompts or perform multi-round local modifications, and their role was primarily restricted to selecting or confirming the final result from the provided candidate outputs. The user roles, AI roles, and specific manipulation methods for each condition are detailed in Table 1.

### 2.4 Experimental procedure

The overall execution workflow for both experiments was strictly administered via a unified online experimental system, following six standardized steps:

*Informed consent*: Upon accessing the system, participants read the study description and the informed consent form; they formally entered the experimental procedure only after clicking to confirm. To minimize the interference of demand characteristics on the experimental results, participants were informed beforehand only that the study focused on “digital art creation experiences and artwork evaluation,” without disclosing the complete serial mediation hypotheses.

*Baseline measurement*: Participants provided demographic information (age, gender, education level) and answered questions assessing control variables, including generative AI usage experience, art/design experience, interest in digital art, and self-rated creativity.

*Random assignment*: Following the baseline measurement, the system’s backend instantaneously invoked a randomization module, automatically allocating participants to the corresponding experimental conditions in a 1:1:1 ratio. The random sequence was system-generated prior to the commencement of the experiment; each participant could be assigned to only one condition and could not foresee their group allocation. During the data collection phase, researchers did not disclose the full serial mediation hypotheses, reiterating only the broad aim of investigating “digital art creation experiences and artwork evaluation” to further reduce demand characteristics.

*Creation task*: Participants read the unified task instructions (theme: “The Symbiosis of Future Cities and Nature”) and executed their artwork conceptualization, generation, curation, or confirmation constrained by the operational permissions of their assigned condition.

*Study 1 (Manipulation of Creation Mode)*: The human-only creation group was required to solely write and submit a description of their artwork concept (no AI participation). The AI-only generation group could only view system-generated works and select the final result. The human-AI co-creation group engaged deeply throughout the process via prompt input, style selection, multi-round generation, and curation.

*Study 2 (Manipulation of AI Involvement Level)*: In the low AI involvement group, the user led the creative decisions while the AI provided limited conceptual suggestions. In the moderate AI involvement group, users retained critical decision-making power over prompts, styles, and curation, engaging in multi-round interactions with the AI. In the high AI involvement group, the AI completed the primary generative process, and the user was only responsible for confirming the final output.

*Psychological measurement*: Once the final artwork was confirmed, the system automatically displayed a preview of the piece. Participants then sequentially completed the psychological ownership scale, perceived agency scale, creative flow scale, perceived creativity scale, aesthetic evaluation scale, and emotional resonance scale.

*Behavioral logging*: Upon task completion, the system backend automatically exported behavioral log data, including creation duration (in seconds), prompt revision counts, AI generation/re-generation counts, style selection counts, final edit counts, and decisions regarding saving and sharing the artwork. Time Control and Exclusion Criteria: To control for the potential confounding effects of task exposure duration on psychological experiences, the system enforced a mandatory minimum dwell time (no less than 60 s for the creation task and 120 s for the psychological measurement). Records with a total completion time below 180 s (indicating rushed or inattentive responding) or exceeding the sample mean by 3 standard deviations (i.e., total duration > 1,500 s, indicating mid-task interruption) were classified as anomalous responses and excluded during the data cleaning phase. The overall execution workflow for both experiments is illustrated in Figure 2.

**Figure 2:**
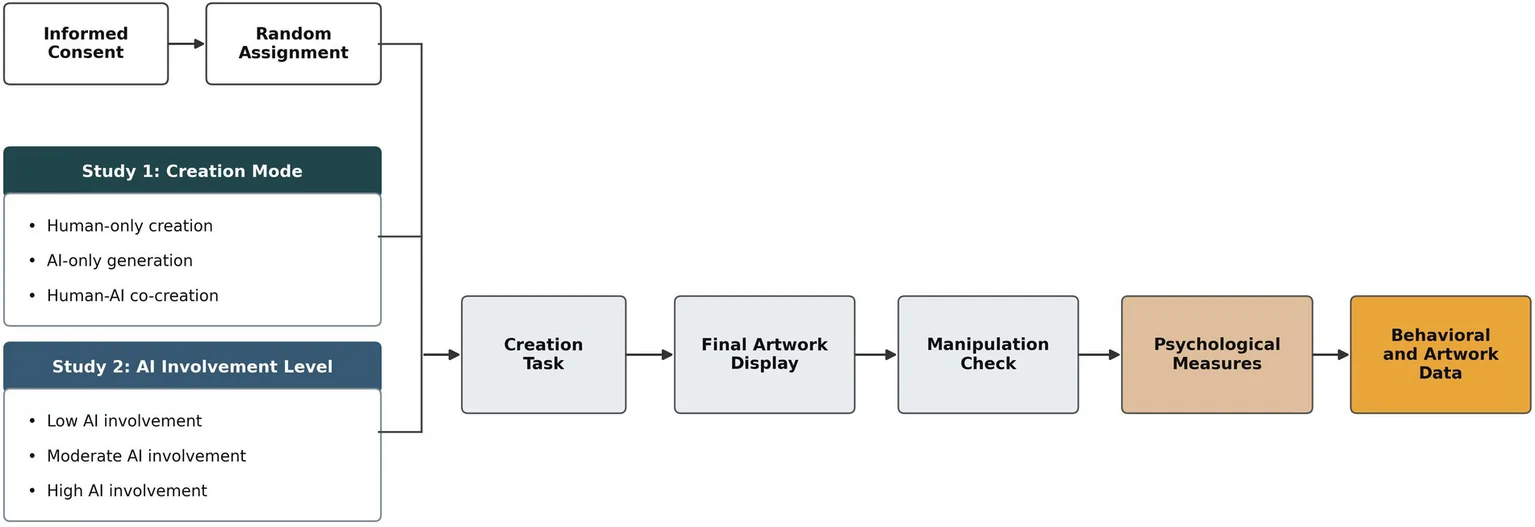
Experimental procedure flowchart.

### 2.5 Measurement instruments

All psychological variables in this study were assessed using a 7-point Likert scale ranging from 1 (“strongly disagree”) to 7 (“strongly agree”). The measurement items were derived from established literature and semantically adapted to ensure contextual appropriateness for generative AI creation scenarios. Specifically, perceived creativity (4 items, based on Amabile, 1982; Runco and Jaeger, 2012) evaluated participants’ subjective assessments of the artwork’s novelty, appropriateness, imagination, and overall creativity. Psychological ownership (4 items, based on Pierce et al., 2001, 2003; Peck and Shu, 2009) measured participants’ perceived sense of belonging, self-extension, and personal investment regarding the artwork. Perceived agency (4 items, based on Synofzik et al., 2008; Moore, 2016; Haggard, 2017) examined participants’ perceived dominance and control over the creative direction and the final output. Creative flow (5 items, based on Csikszentmihalyi, 1990; Hoffman and Novak, 1996; Novak et al., 2000) evaluated the degree of focused immersion and experiential smoothness participants felt during the creation process. Additionally, aesthetic evaluation (3 items, based on Chamberlain et al., 2018; Leder et al., 2004) and emotional resonance (3 items, based on Leder et al., 2004; Silvia, 2005) measured the visual aesthetic appeal of the artwork and the emotional resonance it elicited, respectively.

Because the original scales were in English, a standard back-translation procedure (Brislin, 1970) was used to support cross-linguistic and cultural equivalence. First, two bilingual researchers in human-computer interaction independently translated the English items into Simplified Chinese. Second, an independent bilingual scholar who was blind to the original English questionnaires back-translated the Chinese version into English. Third, the research team compared the back-translated version with the original scales and resolved minor semantic nuances specific to generative-AI art contexts. A pilot test with 30 undergraduate students was conducted before formal deployment to assess the face validity and readability of the Chinese items.

For each variable, the arithmetic mean of the corresponding items was calculated as the analysis index, with higher scores indicating stronger corresponding psychological experiences or evaluations. Sample items and literature sources for the primary variables are detailed in Table 3 (the complete measurement scales are provided in the Supplementary material). Prior to the formal experiment, all items underwent a content validity check by three researchers specializing in psychology and human-computer interaction to ensure semantic accuracy for AI digital art creation. To mitigate common method bias (CMB), items assessing different constructs were presented in a randomized order within the questionnaire; furthermore, control variables were measured prior to the creation task, while the core psychological dependent variables were measured immediately following the task’s completion.

**Table 3:** Measurement variables, sample items, and literature sources.

Variable	Variable name	Item count	Sample item	Literature source
Perceived creativity	perceived_creativity	4	I think this artwork is creative.	Amabile (1982); Runco and Jaeger (2012)
Psychological ownership	psychological_ownership	4	I feel like this artwork belongs to me.	Pierce et al. (2001, 2003); Peck and Shu (2009)
Perceived agency	perceived_agency	4	I feel that I influenced the final artwork.	Synofzik et al. (2008); Moore (2016); Haggard (2017)
Creative flow	creative_flow	5	I was deeply immersed during the creation process.	Csikszentmihalyi (1990); Hoffman and Novak (1996); Novak et al. (2000)
Aesthetic evaluation	aesthetic_evaluation	3	I find this artwork visually appealing.	Chamberlain et al. (2018); Leder et al. (2004)
Emotional resonance	emotional_resonance	3	This artwork elicited an emotional resonance within me.	Leder et al. (2004); Silvia (2005)

### 2.6 Artwork evaluation and behavioral indicators

To supplement the self-report scales and empirically illustrate participants’ behavioral engagement, human-AI interaction patterns, and practical acceptance of the generated artworks, this study collected behavioral process logs and external artwork evaluation data (indicator details are provided in Table 4). Behavioral process data were automatically logged by the experimental system’s backend, encompassing interaction engagement indicators (creation duration in seconds, prompt revision count, AI generation/re-generation count, style selection count, and final edit count) as well as behavioral acceptance indicators (choices regarding saving and sharing the artwork).

**Table 4:** Behavioral indicators and artwork evaluation indicators.

Indicator type	Variable name	Definition
Behavioral data	creation_time_seconds	The total time (in seconds) taken by the participant to complete the creation task
Behavioral data	prompt_revision_count	The number of times the participant modified or re-entered prompts
Behavioral data	ai_generation_count	The number of times the participant triggered AI generation or re-generation
Behavioral data	style_selection_count	The number of times the participant selected or switched visual styles
Behavioral data	final_edit_count	The number of times the participant edited or adjusted the final artwork
Behavioral data	save_choice	Whether the participant opted to save the final artwork
Behavioral data	share_choice	Whether the participant opted to share the final artwork
Artwork rating	novelty	Whether the artwork embodies unique conceptual ideas
Artwork rating	usefulness	Whether the artwork appropriately aligns with the task theme
Artwork rating	aesthetic_quality	Whether the artwork demonstrates strong visual appeal
Artwork rating	expressiveness	Whether the artwork effectively communicates the core theme
Artwork rating	overall_creativity	The comprehensive evaluation of the artwork’s overall creativity

The external evaluation of the artworks followed the Consensual Assessment Technique (CAT; Amabile, 1982), a standard approach in creativity research. The CAT framework posits that independent evaluations by domain-relevant experts provide the most reliable operational criterion for assessing product-level creativity without imposing artificial rating constraints (Kaufman et al., 2009). Three independent evaluators with backgrounds in digital art and visual design, who were blind to the experimental conditions, provided 7-point Likert ratings for the final artworks based on unified rating instructions. The evaluation dimensions included novelty, usefulness/appropriateness, aesthetic quality, expressiveness, and overall creativity. External ratings were used as supplementary artwork-level validation rather than as primary dependent variables. The arithmetic mean of the three evaluators’ scores for each dimension was computed as the final index, and the Intraclass Correlation Coefficient (ICC) was calculated to assess inter-rater reliability. The detailed rating protocol is provided in Supplementary Table S5.

### 2.7 Data analysis

#### 2.7.1 Data screening and quality control

Data cleaning was rigorously executed according to four pre-established quality control criteria:

*Completeness check*: Samples with incomplete creation tasks or missing values in key psychological measurement questionnaires were excluded.

*Attention check*: Two attention-check items were randomly embedded within the psychological questionnaires (e.g., “To confirm that you are reading carefully, please select ‘Somewhat Agree’ for this item”). Participants failing to respond as instructed to either item were classified as invalid samples and excluded.

*Duration anomaly check*: Utilizing the backend behavioral logs, extreme samples characterized by a total response duration of less than 180 s (indicating rushed or inattentive responding) or exceeding the overall mean by three standard deviations (indicating mid-task interruptions) were excluded.

*Outlier and logical check*: Boxplot analyses were performed on the psychological measurement variables to identify and exclude samples exhibiting straight-lining (consecutive identical responses across multiple items) or obvious logical contradictions.

The screening criteria were applied before the confirmatory analyses. Table 5 reports the initial responses, exclusions by study, final analytic samples, and retention rates.

**Table 5:** Data screening and sample retention.

Data source	Original sample size	Reasons for exclusion	Excluded sample size	Final valid sample size	Efficiency
Study 1	326	Incomplete procedures, failed attention checks, abnormal response duration, missing key variables, etc.	26	300	92.0%
Study 2	329	Incomplete procedures, failed attention checks, abnormal response duration, missing key variables, etc.	29	300	91.2%

#### 2.7.2 Statistical analysis and modeling strategy

Data cleaning, statistical testing, and mediation modeling for this study were executed within the Python (3.10+) data science and statistical analysis ecosystem. The specific analytical workflow was as follows:

First, Cronbach’s α coefficients were calculated for all scales to assess internal consistency reliability. Composite Reliability (CR) and Average Variance Extracted (AVE) were calculated based on the standardized factor loadings derived from Confirmatory Factor Analysis (CFA) to evaluate convergent validity. Additionally, the semopy library was utilized to construct a multi-construct CFA model, reporting model fit indices including *χ*^2^/df, CFI, TLI, RMSEA, and SRMR. These indices were evaluated against the standard structural cutoff criteria recommended by Hu and Bentler (1999).

Second, the means (M), standard deviations (SD), and Pearson correlation matrices for the primary variables were reported.

Third, Harman’s single-factor test was employed to assess the potential impact of common method bias. In addition, procedural remedies (e.g., temporal separation and randomized item presentation) were adopted to mitigate common method variance (Podsakoff et al., 2003).

Fourth, one-way analysis of variance (ANOVA) was used to test condition differences in psychological outcomes, aesthetic evaluation, emotional resonance, and behavioral indicators. Distributions and Levene’s tests were examined before group comparisons. When homogeneity of variance was supported, standard ANOVA and Tukey’s HSD comparisons were used. When it was violated, Welch’s ANOVA and Games-Howell comparisons were used. We report partial eta squared as the omnibus effect-size estimate.

Fifth, the serial mediation effects were tested utilizing Ordinary Least Squares (OLS) regression coupled with 5,000 Bootstrap resamplings. Using the AI-only generation group as the reference and human-AI co-creation as the core predictor variable, a serial mediation model was constructed: “Human-AI Co-creation → Psychological Ownership → Perceived Agency → Creative Flow → Perceived Creativity” (conceptually aligning with PROCESS Model 6 logic). This analytical framework follows the bias-corrected bootstrap approach for serial multiple mediation described by Hayes (2018). An indirect effect was deemed statistically significant if the 95% bias-corrected Bootstrap confidence interval did not cross zero.

Sixth, robustness models included age, gender, AI-use experience, art experience, interest in digital art, and self-rated creativity as covariates. For Study 2, the omnibus ANOVA and comparisons among the three prespecified workflows were treated as the primary tests. To examine the observed non-monotonic pattern, condition was coded as an ordered, mean-centered variable (−1 = low involvement, 0 = moderate involvement, +1 = high involvement), and the quadratic term was defined as the square of that code. The negative quadratic coefficient was interpreted together with the observed peak in the moderate condition. Because the conditions simultaneously changed several interaction permissions, this supplementary test is not interpreted as a continuous dose–response function. All tests were two-tailed with alpha = 0.05. Random assignment supports causal interpretation of condition effects, but the indirect paths among concurrently measured post-task variables are interpreted as associations consistent with the proposed process.

## 3 Results

### 3.1 Data screening

Table 5 summarizes sample screening and retention; the prespecified exclusion criteria are defined in Section 2.7.1.

### 3.2 Manipulation check

To verify the effectiveness of the experimental manipulations, this study examined the levels of AI involvement and human-AI co-creation across different conditions. Because the formal experimental procedure did not include an independent subjective self-report manipulation check scale, this study relied primarily on objective behavioral logs recorded by the system backend to conduct the manipulation check. Specifically, the “number of system generations/displays” served as the behavioral proxy indicator for the level of AI involvement, while the “sum of prompt modifications, style selections, and final edits” served as the behavioral proxy indicator for the level of co-creation. These indicators provide evidence of manipulation at the level of actual interaction behaviors, though they are not directly equivalent to participants’ subjectively perceived degree of AI dominance or user control; therefore, interpretations of these metrics are strictly confined to behavioral evidence of manipulation.

The Study 1 conditions differed in the number of system generations or displays, *F*(2, 297) = 90.131, *p* < 0.001, partial eta squared = 0.378. The human-only condition had the lowest count (M = 1.240, SD = 1.138), the AI-only condition had the highest count (M = 4.360, SD = 2.052), and the co-creation condition was also high (M = 3.840, SD = 1.947). The conditions also differed on the co-creation behavioral proxy, *F*(2, 297) = 88.731, *p* < 0.001, partial eta squared = 0.374. The co-creation condition had the highest score (M = 8.270, SD = 3.275), followed by the human-only (M = 4.640, SD = 2.695) and AI-only conditions (M = 3.200, SD = 2.256).

In Study 2, the conditions differed in AI-generation counts, *F*(2, 297) = 108.467, *p* < 0.001, partial eta squared = 0.422. The low-involvement condition had the lowest count (M = 0.950, SD = 0.999); the moderate (M = 3.940, SD = 2.088) and high conditions (M = 4.220, SD = 1.931) had higher counts. The conditions also differed on the co-creation behavioral proxy, *F*(2, 297) = 77.644, *p* < 0.001, partial eta squared = 0.343. The moderate condition had the highest score (M = 8.350, SD = 3.873), followed by the low (M = 4.270, SD = 2.704) and high conditions (M = 3.420, SD = 2.128). These behavioral indicators confirm that participants encountered different workflows, but they do not directly measure perceived AI dominance or user control (see Table 6).

**Table 6:** Manipulation check results.

Study	Manipulation Check Variable	Condition 1 (M ± SD)	Condition 2 (M ± SD)	Condition 3 (M ± SD)	F	*p*	$\eta_{\rho }^{2}$
Study 1	AI Involvement Proxy: System Generation/Display Count	Human-only 1.240 ± 1.138	AI-only 4.360 ± 2.052	Co-creation 3.840 ± 1.947	90.131	<0.001	0.378
Study 1	Co-creation Proxy: Prompt/Style/Edit Count	Human-only 4.640 ± 2.695	AI-only 3.200 ± 2.256	Co-creation 8.270 ± 3.275	88.731	<0.001	0.374
Study 2	AI Involvement Proxy: System Generation/Display Count	Low AI 0.950 ± 0.999	Moderate AI 3.940 ± 2.088	High AI 4.220 ± 1.931	108.467	<0.001	0.422
Study 2	Co-creation Proxy: Prompt/Style/Edit Count	Low AI 4.270 ± 2.704	Moderate AI 8.350 ± 3.873	High AI 3.420 ± 2.128	77.644	<0.001	0.343

### 3.3 Reliability and validity analysis

Three independent evaluators rated the final artworks on novelty, usefulness, aesthetic quality, expressiveness, and overall creativity. Inter-rater reliability was high (ICC = 0.87, *p* < 0.001), supporting aggregation across raters. These ratings provide a product-level assessment that differs conceptually from participants’ self-reported perceived creativity. The two measures were analyzed separately and were not treated as interchangeable. Because perceived creativity was the prespecified primary outcome, the external ratings were used as supplementary validation. Analysis of the external ratings showed condition differences in overall creativity in Study 1, *F*(2, 297) = 12.68, *p* < 0.001, partial eta squared = 0.079, and Study 2, *F*(2, 297) = 20.35, *p* < 0.001, partial eta squared = 0.121. In Study 1, the co-creation condition received higher external ratings (M = 4.81, SD = 0.91) than the human-only (M = 4.16, SD = 0.95) and AI-only conditions (M = 4.34, SD = 1.02). In Study 2, the moderate-involvement workflow received the highest external ratings (M = 4.96, SD = 0.88), exceeding the low- (M = 4.12, SD = 0.98) and high-involvement workflows (M = 4.41, SD = 1.03). Although the condition differences were less pronounced than those in participants’ self-reported ratings, the overall pattern was consistent across the two forms of assessment. Supplementary Table S5 reports the condition-level external-rating results.

The Cronbach’s α coefficients for perceived creativity, psychological ownership, perceived agency, creative flow, aesthetic evaluation, and emotional resonance were 0.876, 0.874, 0.883, 0.898, 0.820, and 0.834, respectively, all exceeding the 0.80 threshold. Composite Reliability (CR) values ranged from 0.893 to 0.925, and Average Variance Extracted (AVE) values ranged from 0.711 to 0.751. The standardized factor loadings for the items of each construct were consistently high, indicating satisfactory convergent validity for the primary variables. Subsequent confirmatory factor analysis (CFA) revealed that the six-factor measurement model fit the data well: χ^2^/df = 1.139, CFI = 0.996, TLI = 0.996, RMSEA = 0.015 (90% CI [0.000, 0.026]), and SRMR = 0.035. Discriminant validity was evaluated using both the Fornell–Larcker criterion and the Heterotrait-Monotrait (HTMT) ratio. As presented in Table 7, the square root of the AVE for each construct (ranging from 0.843 to 0.867) exceeded its highest bivariate correlation with any other construct (max *r* = 0.600). Furthermore, all HTMT values remained well below the 0.85 threshold (ranging from 0.201 to 0.685), establishing satisfactory discriminant validity.

**Table 7:** Descriptive statistics, correlations, and discriminant validity (Fornell–Larcker & HTMT).

Variable	M	SD	1	2	3	4	5	6
1. Perceived creativity	4.170	1.260	**(0.853)**	[0.632]	[0.628]	[0.685]	[0.458]	[0.424]
2. Psychological ownership	4.278	1.247	0.565***	**(0.852)**	[0.615]	[0.568]	[0.218]	[0.457]
3. Perceived agency	4.070	1.271	0.564***	0.555***	**(0.860)**	[0.604]	[0.201]	[0.359]
4. Creative flow	4.156	1.259	0.600***	0.503***	0.541***	**(0.843)**	[0.402]	[0.501]
5. Aesthetic evaluation	4.273	1.251	0.395***	0.190**	0.174**	0.348***	**(0.858)**	[0.298]
6. Emotional resonance	4.237	1.268	0.369***	0.398***	0.314***	0.439***	0.256***	**(0.867)**

*N* = 600. Diagonal values in bold parentheses are the square roots of the AVE. Values below the diagonal are Pearson correlations (**p < 0.01, ***p < 0.001). Values above the diagonal in brackets are Heterotrait-Monotrait (HTMT) ratios.

Furthermore, because the core psychological variables in this study were collected via self-report scales, there is a potential for common method bias (CMB). This issue was statistically assessed using Harman’s single-factor test. The results of the unrotated exploratory factor analysis showed that the first principal component accounted for only 32.18% of the total variance, well below the critical threshold of 50%. This suggests that common method bias was unlikely to substantially distort the main findings. The reliability and validity results are summarized in Table 8.

**Table 8:** Reliability and validity test results of measurement scales.

Variable	Item count	Cronbach’s α	CR	AVE	Factor loading range
Perceived creativity	4	0.876	0.915	0.728	0.845–0.858
Psychological ownership	4	0.874	0.914	0.726	0.836–0.866
Perceived agency	4	0.883	0.919	0.740	0.844–0.871
Creative flow	5	0.898	0.925	0.711	0.820–0.859
Aesthetic evaluation	3	0.820	0.893	0.736	0.842–0.867
Emotional resonance	3	0.834	0.901	0.751	0.862–0.874

### 3.4 Descriptive statistics and correlation analysis

The means (M), standard deviations (SD), and correlation coefficients of the primary variables are presented in Table 7. Overall, perceived creativity exhibited moderate positive correlations with psychological ownership (*r* = 0.565), perceived agency (*r* = 0.564), and creative flow (*r* = 0.600). Perceived creativity was also positively correlated with aesthetic evaluation (*r* = 0.395) and emotional resonance (*r* = 0.369). Psychological ownership was highly correlated with perceived agency (*r* = 0.555) and creative flow (*r* = 0.503), while perceived agency also showed a positive correlation with creative flow (*r* = 0.541). These findings provide preliminary empirical support for the subsequent serial mediation analysis.

### 3.5 Study 1: impact of creation mode on psychological variables

Study 1 showed a condition effect on perceived creativity, *F*(2, 297) = 34.812, *p* < 0.001, partial eta squared = 0.190. The human-AI co-creation condition had the highest mean (M = 4.645, SD = 1.090), followed by human-only creation (M = 4.272, SD = 1.270) and AI-only generation (M = 3.315, SD = 1.121). Post-hoc comparisons showed that the AI-only condition was lower than both the co-creation and human-only conditions.

Creation mode also affected psychological ownership, *F*(2, 297) = 69.828, *p* < 0.001, partial eta squared = 0.320; perceived agency, *F*(2, 297) = 63.000, *p* < 0.001, partial eta squared = 0.298; and creative flow, *F*(2, 297) = 42.547, *p* < 0.001, partial eta squared = 0.223. Psychological ownership was highest in the human-only condition (M = 4.782, SD = 1.146), followed by co-creation (M = 4.600, SD = 1.042) and AI-only generation (M = 3.143, SD = 1.035). Perceived agency was highest in co-creation (M = 4.593, SD = 1.120), followed by human-only creation (M = 4.372, SD = 1.114) and AI-only generation (M = 2.973, SD = 1.087). Creative flow was similarly high in the co-creation (M = 4.492, SD = 1.086) and human-only conditions (M = 4.430, SD = 1.242), and lower in the AI-only condition (M = 3.164, SD = 1.112).

Creation mode also affected aesthetic evaluation, *F*(2, 297) = 6.002, *p* = 0.0028, partial eta squared = 0.039, and emotional resonance, *F*(2, 297) = 14.099, *p* < 0.001, partial eta squared = 0.087. The co-creation condition had higher aesthetic ratings than the AI-only condition. Emotional resonance was lower in the AI-only condition than in both the co-creation and human-only conditions. Figure 3 displays the group means, and Table 9 reports the ANOVA results.

**Figure 3:**
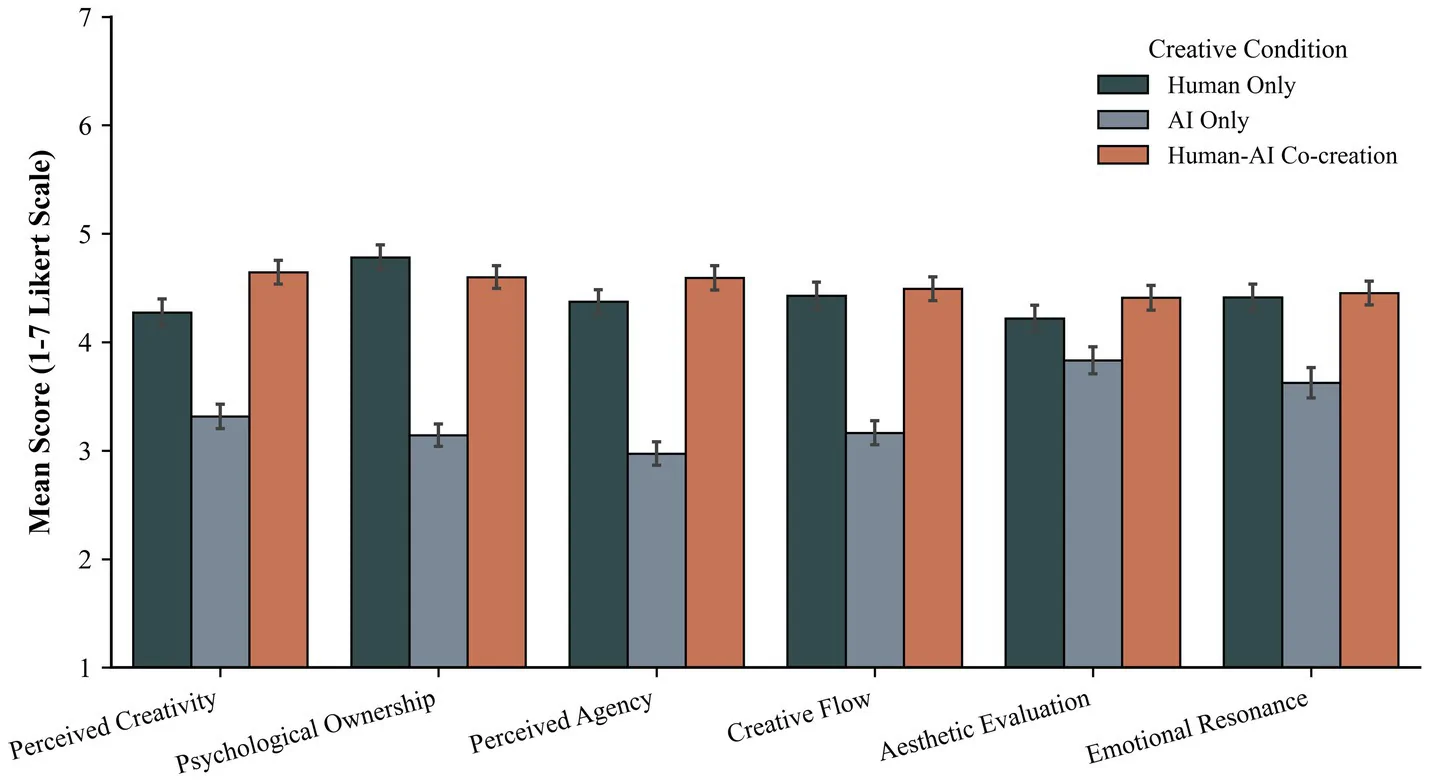
Impact of creation mode on primary psychological variables.

**Table 9:** ANOVA results of creation mode in study 1.

Dependent variable	Human-only M (SD)	AI-only M (SD)	Co-creation M (SD)	F	*p*	$\eta_{\rho }^{2}$	*Post-hoc*
Perceived Creativity	4.272 ± 1.270	3.315 ± 1.121	4.645 ± 1.090	34.812	<0.001	0.190	AI-only < co-creation; AI-only < human-only
Psychological Ownership	4.782 ± 1.146	3.143 ± 1.035	4.600 ± 1.042	69.828	<0.001	0.320	AI-only < co-creation; AI-only < human-only
Perceived Agency	4.372 ± 1.114	2.973 ± 1.087	4.593 ± 1.120	63.000	<0.001	0.298	AI-only < co-creation; AI-only < human-only
Creative Flow	4.430 ± 1.242	3.164 ± 1.112	4.492 ± 1.086	42.547	<0.001	0.223	AI-only < co-creation; AI-only < human-only
Aesthetic Evaluation	4.220 ± 1.203	3.833 ± 1.251	4.410 ± 1.142	6.002	0.0028	0.039	Co-creation > AI-only
Emotional Resonance	4.413 ± 1.211	3.627 ± 1.397	4.453 ± 1.098	14.099	<0.001	0.087	AI-only < co-creation; AI-only < human-only

### 3.6 Study 1: serial mediation effect testing

To further examine the psychological mechanisms through which human-AI co-creation influences perceived creativity, this study constructed a serial mediation model: “Human-AI Co-creation → Psychological Ownership → Perceived Agency → Creative Flow → Perceived Creativity.” The AI-only generation group was designated as the reference group, and human-AI co-creation was set as the core predictor variable. Bootstrap testing results revealed a direct effect of 0.2223, 95% CI = [−0.1152, 0.5439]. Because the confidence interval includes zero, the direct effect of human-AI co-creation on perceived creativity is non-significant after incorporating the mediator variables. The total indirect effect was 1.1077, 95% CI = [0.8226, 1.4392], indicating that the psychological mechanism pathways exerted a significant mediating role.

Regarding the specific indirect pathways, the individual mediating effect of psychological ownership was significant (Effect = 0.3900, 95% CI = [0.1695, 0.6575]), the individual mediating effect of perceived agency was significant (Effect = 0.1980, 95% CI = [0.0814, 0.3510]), and the individual mediating effect of creative flow was significant (Effect = 0.1389, 95% CI = [0.0356, 0.2687]). Furthermore, the pathway of “Psychological Ownership → Perceived Agency → Perceived Creativity” was significant (Effect = 0.1647, 95% CI = [0.0716, 0.2757]), the pathway of “Psychological Ownership → Creative Flow → Perceived Creativity” was significant (Effect = 0.1241, 95% CI = [0.0529, 0.2143]), and the pathway of “Perceived Agency → Creative Flow → Perceived Creativity” was significant (Effect = 0.0502, 95% CI = [0.0143, 0.1032]). Crucially, the complete serial mediation pathway, “Human-AI Co-creation → Psychological Ownership → Perceived Agency → Creative Flow → Perceived Creativity,” was also significant (Effect = 0.0418, 95% CI = [0.0122, 0.0834]). These results are presented in Figure 4 and Table 10.

**Figure 4:**
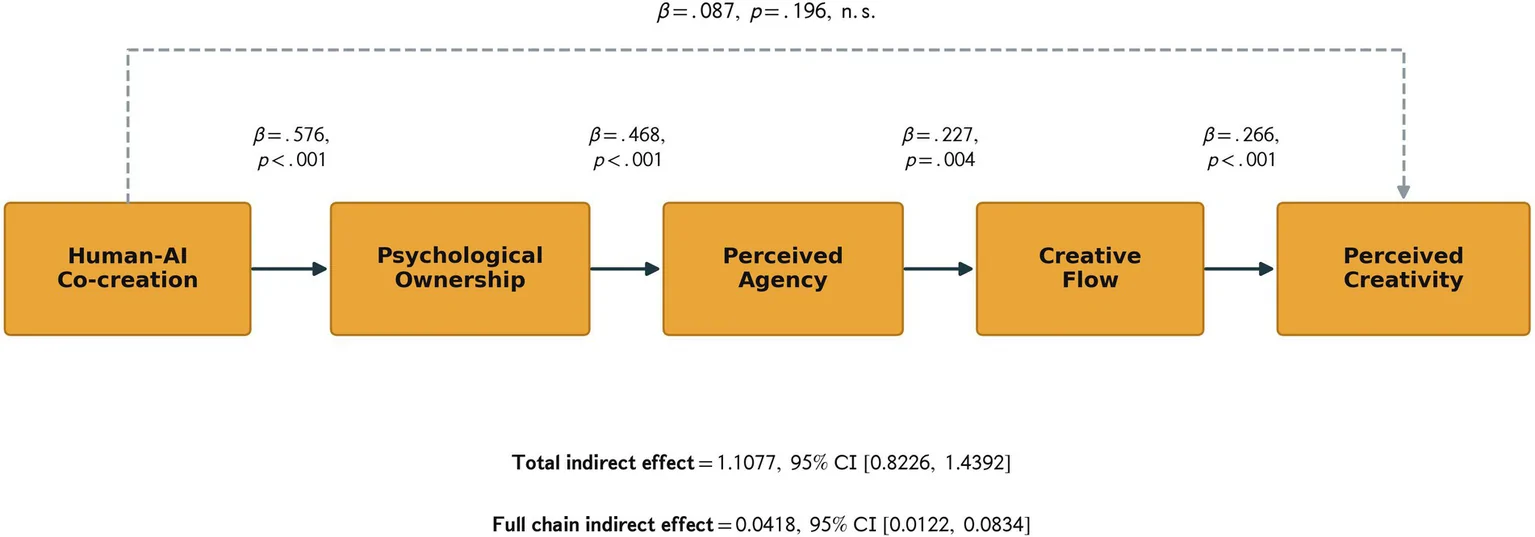
Standardized path coefficients of the serial mediation model in Study 1.

**Table 10:** Direct and indirect effects of the serial mediation model.

Effect pathway	Effect	BootSE	95% CI lower limit	95% CI upper limit	Conclusion
Direct effect	0.2223	0.1682	−0.1152	0.5439	Non-significant
Total indirect effect	1.1077	0.1570	0.8226	1.4392	Significant
Human-AI Co-creation → Psychological Ownership → Perceived Creativity	0.3900	0.1236	0.1695	0.6575	Significant
Human-AI Co-creation → Perceived Agency → Perceived Creativity	0.1980	0.0691	0.0814	0.3510	Significant
Human-AI Co-creation → Creative Flow → Perceived Creativity	0.1389	0.0592	0.0356	0.2687	Significant
Human-AI Co-creation → Psychological Ownership → Perceived Agency → Perceived Creativity	0.1647	0.0521	0.0716	0.2757	Significant
Human-AI Co-creation → Psychological Ownership → Creative Flow → Perceived Creativity	0.1241	0.0415	0.0529	0.2143	Significant
Human-AI Co-creation → Perceived Agency → Creative Flow → Perceived Creativity	0.0502	0.0226	0.0143	0.1032	Significant
Human-AI Co-creation → Psychological Ownership → Perceived Agency → Creative Flow → Perceived Creativity	0.0418	0.0182	0.0122	0.0834	Significant

Unstandardized indirect and direct path effects with 5,000 bootstrap resamplings are reported.

### 3.7 Study 2: impact of AI involvement levels on perceived creativity

Study 2 showed a condition effect on perceived creativity, *F*(2, 297) = 57.204, *p* < 0.001, partial eta squared = 0.278. The moderate-involvement workflow produced the highest mean (M = 5.155, SD = 1.025), exceeding both the low-involvement (M = 3.985, SD = 1.077) and high-involvement workflows (M = 3.647, SD = 1.035).

The Study 2 conditions also differed in psychological ownership, *F*(2, 297) = 19.464, *p* < 0.001, partial eta squared = 0.116; perceived agency, *F*(2, 297) = 51.389, *p* < 0.001, partial eta squared = 0.257; and creative flow, *F*(2, 297) = 42.597, *p* < 0.001, partial eta squared = 0.223. The moderate workflow had the highest mean on all three variables. The high-involvement workflow had the lowest psychological ownership (M = 3.875, SD = 1.179), perceived agency (M = 3.425, SD = 1.004), and creative flow (M = 3.692, SD = 1.001).

The Study 2 conditions also differed in aesthetic evaluation, *F*(2, 297) = 10.079, *p* < 0.001, partial eta squared = 0.064, and emotional resonance, *F*(2, 297) = 14.890, *p* < 0.001, partial eta squared = 0.091. The moderate workflow produced the highest mean on both outcomes. Figure 5 displays perceived creativity across the three workflows, and Table 11 reports the ANOVA results. Because each condition bundled several interaction permissions, this pattern is described as non-monotonic across the tested workflows rather than as a general continuous inverted-U function.

**Figure 5:**
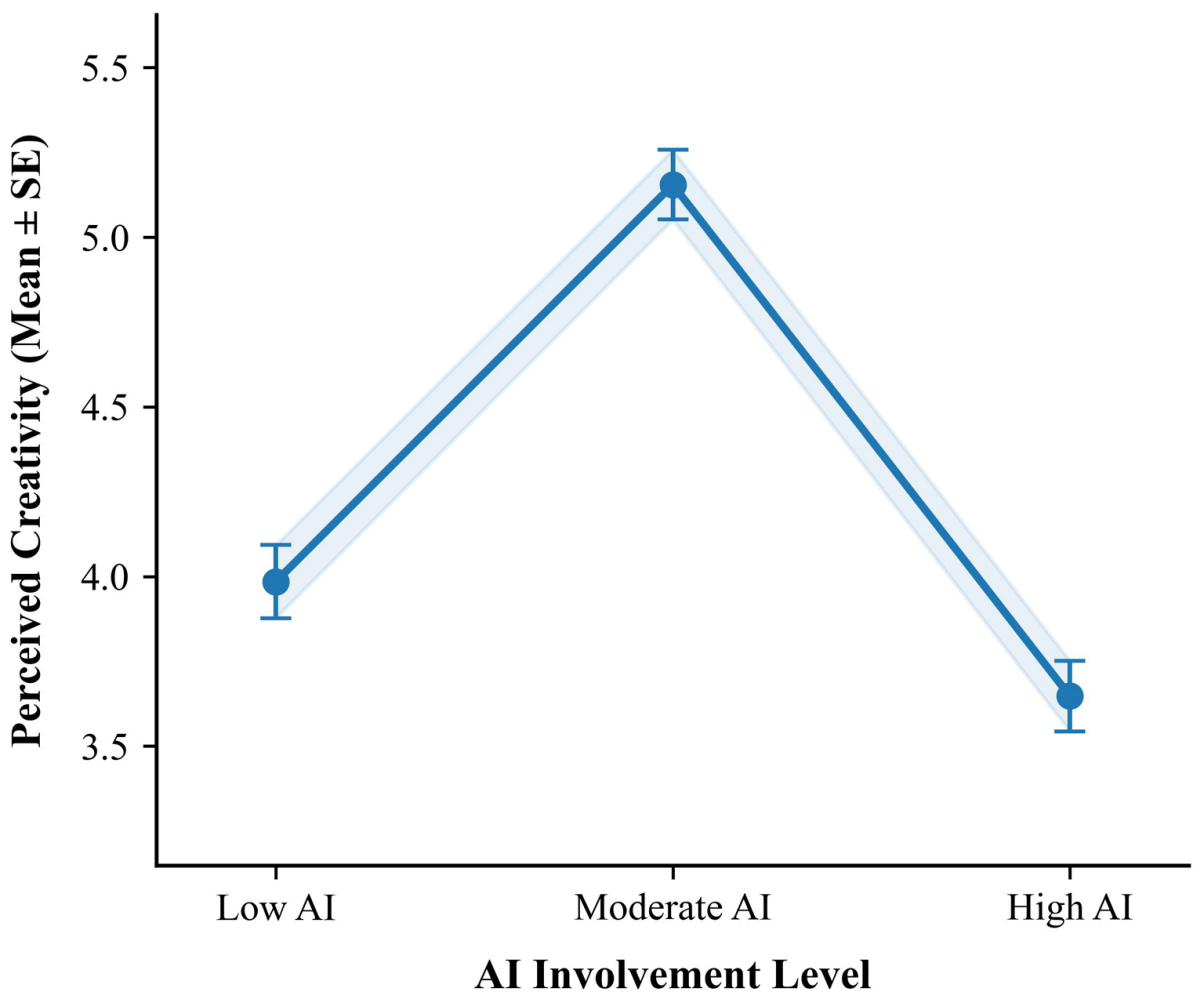
Impact of AI involvement levels on perceived creativity.

**Table 11:** ANOVA results of AI involvement levels in study 2.

Dependent Variable	Low M (SD)	Moderate M (SD)	High M (SD)	F	*p*	η_p_^2^	*Post-hoc*
Perceived creativity	3.985 ± 1.077	5.155 ± 1.025	3.647 ± 1.035	57.204	<0.001	0.278	Moderate > low, high
Psychological ownership	4.407 ± 1.157	4.860 ± 1.008	3.875 ± 1.179	19.464	<0.001	0.116	All pairwise differences significant
Perceived agency	4.122 ± 1.093	4.935 ± 1.063	3.425 ± 1.004	51.389	<0.001	0.257	All pairwise differences significant
Creative flow	4.108 ± 1.178	5.052 ± 1.015	3.692 ± 1.001	42.597	<0.001	0.223	All pairwise differences significant
Aesthetic evaluation	4.113 ± 1.257	4.840 ± 1.223	4.220 ± 1.227	10.079	0.0001	0.064	Moderate > low, high
Emotional resonance	4.153 ± 1.102	4.823 ± 1.201	3.950 ± 1.245	14.890	<0.001	0.091	Moderate > low, high

### 3.8 Robustness checks

To test the stability of the findings, this study further incorporated control variables, including age, art experience, AI usage experience, interest in digital art, and self-rated creativity. The results indicated that, without the inclusion of control variables, human-AI co-creation significantly and positively predicted perceived creativity relative to AI-only generation, β = 1.3300, SE = 0.1563, *p* < 0.001, 95% CI = [1.0217, 1.6383]. After controlling for age and art experience, this effect remained significant, β = 1.3236, SE = 0.1551, *p* < 0.001, 95% CI = [1.0177, 1.6295]. Furthermore, after controlling for AI usage experience, interest in digital art, and self-rated creativity, the effect maintained its significance, β = 1.3528, SE = 0.1520, *p* < 0.001, 95% CI = [1.0530, 1.6526].

In Study 2, the quadratic term for the ordered condition code was negative, beta = −1.3428, SE = 0.1200, *p* < 0.001, 95% CI [−1.5791, −1.1065]. This supplementary result was consistent with the higher perceived-creativity mean in the moderate workflow. It does not establish a continuous dose–response relation because the three conditions differed in several design features. Table 12 reports the covariate-adjusted models.

**Table 12:** Robustness check results controlling for covariates.

Model	Control variables	Core predictor variable	Dependent variable	β/Effect	SE	*p*	95% CI
Model 1	None	Human-AI Co-creation vs. AI-only	Perceived creativity	1.3300	0.1563	<0.001	[1.0217, 1.6383]
Model 2	Age, Art Experience	Human-AI Co-creation vs. AI-only	Perceived creativity	1.3236	0.1551	<0.001	[1.0177, 1.6295]
Model 3	Age, Art Exp., AI Usage Exp., Digital Art Interest, Self-rated Creativity	Human-AI Co-creation vs. AI-only	Perceived creativity	1.3528	0.1520	<0.001	[1.0530, 1.6526]
Model 4	Age, Art Exp., AI Usage Exp., Digital Art Interest, Self-rated Creativity	AI Involvement (Quadratic Term)	Perceived creativity	−1.3428	0.1200	<0.001	[−1.5791, −1.1065]

## 4 Discussion

### 4.1 General discussion

This study examined why participation in generative-AI art may shape perceived creativity. Across the tested conditions, favorable creativity judgments were associated with users’ sense that they had contributed to the work, could influence its development, and remained engaged during creation. The findings therefore shift attention from the source label of an image alone to the psychological experience created by a particular human-AI workflow.

Study 1 showed that human-AI co-creation produced higher perceived creativity, psychological ownership, perceived agency, and creative flow than AI-only generation. Human-only creation retained the highest psychological ownership, whereas co-creation produced the highest perceived agency and perceived creativity. This pattern suggests that AI assistance need not reduce subjective creativity when users retain consequential input and selection opportunities. It is consistent with accounts of creativity-support tools that emphasize exploration, feedback, and expressive control (Shneiderman, 2007; Lubart, 2005).

The indirect-effect analysis was consistent with the proposed sequence from psychological ownership to perceived agency, creative flow, and perceived creativity. The direct effect became non-significant after the mediators were entered, while the complete serial indirect effect excluded zero. This result does not establish temporal causality among the mediators because they were measured concurrently after the task. It instead identifies an internally coherent pattern in which ownership, agency, and flow jointly account for condition-related differences in perceived creativity.

Study 2 showed that the moderate workflow produced more favorable ratings than both the low- and high-involvement workflows. Limited assistance may have provided insufficient generative expansion or feedback. The high-involvement workflow, by restricting prompt revision and local modification, may have reduced users’ sense of contribution and control. The moderate condition combined generative support with repeated choices over prompts, styles, and output selection, which may have better supported agency and flow. These explanations remain provisional because the conditions bundled AI output, user effort, editing permissions, and decision authority. Novelty, task difficulty, and familiarity with generative AI may also have contributed to the observed differences.

### 4.2 Theoretical contributions

The first contribution is to reposition perceived creativity in generative-AI art as a judgment shaped by both the output and the collaborative process. Prior work has shown that authorship labels, authenticity judgments, and source biases affect responses to AI art (Chamberlain et al., 2018; Bellaiche et al., 2023; Horton et al., 2023). The present experiments extend this literature by comparing users who encountered different roles and interaction permissions during creation. This process perspective explains why visually generated outputs may be evaluated differently when users experience different degrees of participation.

The second contribution is the theoretically ordered integration of psychological ownership, perceived agency, and creative flow. These constructs are often discussed separately, yet the indirect-effect estimates were consistent with a process in which personal investment relates to control, control relates to immersion, and immersion relates to perceived creativity. This model does not prove a temporal causal chain, but it provides a testable account of how self-referential experiences may enter creativity judgments during human-AI co-creation.

The third contribution is to show that human-AI collaboration cannot be summarized by the amount of automation alone. The moderate workflow was associated with higher perceived creativity than the low- and high-involvement workflows. This result extends human-AI interaction research by identifying the distribution of meaningful choices and feedback opportunities as a plausible boundary condition (Amershi et al., 2019; Rezwana and Maher, 2023). The contribution is limited to the three workflows tested and should not be interpreted as a universal optimal percentage of AI involvement.

### 4.3 Practical implications

The results support interfaces that preserve consequential user actions throughout generation. Prompt revision, side-by-side comparison, local editing, version history, and reversible selection allow users to see how their decisions shape the final work. Systems should also expose which inputs produced each output and provide checkpoints before major automated changes. These features directly target the ownership and agency differences observed across the experimental conditions.

Automation should be adjustable by task and user rather than fixed at the highest available level. A useful workflow can offer suggestions or draft variants while reserving decisions about concept, style, revision, and final selection for the user. The present results do not establish one universal automation level, but they indicate that removing meaningful choices may reduce perceived agency and creative flow. Designers should therefore evaluate AI systems with process measures, such as perceived control and contribution, alongside output quality and completion time.

In creative education, generative AI can be used as a documented collaborator. Learners can submit prompt histories, explain why alternatives were rejected, identify their own modifications, and compare self-assessed creativity with independent evaluation of the final work. This structure makes conceptual judgment and revision visible rather than rewarding one-click output production. It also connects AI-assisted practice with established work on collaborative design and creative confidence (Agboola and Yassin, 2025; Dildabek and Tsakalerou, 2026).

### 4.4 Limitations and future research

First, the experiment used one digital-art theme and did not hold the form of creative production fully constant across Study 1 conditions. The human-only condition submitted an artwork concept, whereas AI-enabled conditions involved viewing or generating images. Differences in task form, output concreteness, and required effort may therefore contribute to condition effects. Future studies should compare workflows that produce equivalent final artifacts and should replicate the model across visual design, writing, music, and professional creative tasks.

Second, manipulation checks relied on behavioral logs, including generation counts, prompt revisions, and style selections. These measures verify differences in interaction behavior but do not directly measure perceived AI dominance, autonomy, or control. Future experiments should combine behavioral logs with manipulation-check items that are distinct from the proposed mediators.

Third, the low-, moderate-, and high-involvement conditions bundled several features: AI output, prompt authority, regeneration opportunities, editing permissions, and final-selection control. Their effects cannot be attributed to a single continuous quantity of AI involvement. Factorial experiments that manipulate these features separately are needed to identify which design element produces changes in ownership, agency, flow, and creativity judgments.

Fourth, the sample consisted mainly of students and participants with low-to-moderate art and AI-use experience. Short-term exposure to a single system may not represent sustained professional practice, and prior AI use is not equivalent to AI literacy. Replication should recruit professional artists and designers, measure technical and critical AI literacy, and examine whether expertise changes responses to automation and control.

Fifth, random assignment supports causal conclusions about the tested conditions, but not about the ordering of the mediators. Psychological ownership, perceived agency, creative flow, and perceived creativity were measured concurrently after the task. The serial mediation estimates should therefore be interpreted as a theoretically ordered indirect-effect model. Process tracing, repeated measurement during creation, or experimental manipulation of individual mediators would provide stronger evidence about temporal sequence.

Sixth, perceived creativity was a self-reported judgment and may contain self-enhancement, ownership, or demand-related components. Independent raters provided supplementary product-level assessments, but the present manuscript does not treat those ratings as equivalent to the primary outcome. Future studies should preregister both self-assessed and externally assessed creativity as distinct outcomes and test when they converge or diverge, especially considering the potential impacts of cognitive offloading and inflated self-beliefs in AI-assisted workflows (Tong, 2026; Tsakalerou et al., 2026).

Seventh, novelty effects, perceived task difficulty, familiarity with generative AI, and prior creative experience remain plausible alternative explanations. Covariate adjustment for prior experience reduces some observable imbalance but cannot isolate these mechanisms. Follow-up experiments should manipulate novelty and task difficulty directly, include experienced and novice users, and examine repeated use over longer periods.

## 5 Conclusion

Across two between-subjects experiments, human-AI co-creation produced more favorable perceived-creativity judgments than AI-only generation, and the condition difference was statistically consistent with a sequence involving psychological ownership, perceived agency, and creative flow. Among the three Study 2 workflows, the moderate condition produced the highest perceived-creativity ratings. The central implication is that generative support is most likely to be experienced as creative when users retain meaningful influence over the work. This conclusion concerns subjective creativity judgments in the tested digital-art tasks; it does not establish a universal automation optimum or equivalent gains in independently assessed creative performance.

## Data Availability

The original contributions presented in the study are included in the article/Supplementary material, further inquiries can be directed to the corresponding author.
