# Supplementary material for: The impact of human-AI co-creation on perceived creativity in generative AI art: a serial mediation mechanism of psychological ownership, perceived agency, and creative flow
Source: Front Psychol. 2026 Oct 9;17:1986050. doi: 10.3389/fpsyg.2026.1986050 (PMC13690522; doi:10.3389/fpsyg.2026.1986050)
Supplement: Supplementary file 1 [file Supplementary_file_1.DOCX]

**Supplementary Material**

*The Impact of Human-AI Co-creation on Perceived Creativity in Generative AI Art: A Serial Mediation Mechanism of Psychological Ownership, Perceived Agency, and Creative Flow*

# 1. Measurement Instruments

All core variables were measured using 7-point Likert scales unless otherwise specified (1 = strongly disagree, 7 = strongly agree).

## Table S1. Complete questionnaire items

| Construct | Item No. | Items | Source |
| --- | --- | --- | --- |
| Perceived Creativity | PC1  PC2  PC3  PC4 | I think this work is creative.  I think this work is novel.  I think this work appropriately expresses the task theme.  I think this work is imaginative. | Amabile, 1982;  Runco and Jaeger, 2012 |
| Psychological Ownership | PO1  PO2  PO3  PO4 | I feel that this work belongs to me.  I feel that this work reflects my ideas.  I have a strong sense of ownership toward this work.  I feel that I invested something personal in this work. | Pierce et al., 2001, 2003;  Peck and Shu, 2009 |
| Perceived Agency | PA1  PA2  PA3  PA4 | I feel that I was able to influence the generation direction of the work.  I feel that the final work was influenced by my choices and actions.  I feel that I played a leading role in the creative process.  I feel that the outcome of the work was related to my actions. | Synofzik et al., 2008;  Moore, 2016;  Haggard, 2017 |
| Creative Flow | CF1  CF2  CF3  CF4  CF5 | I remained focused during the creative process.  I felt that the creative process was smooth.  I felt engaged during the creative process.  I felt that the creative task was appropriately challenging.  I felt enjoyment or immersion during the creative process. | Csikszentmihalyi, 1990;  Hoffman and Novak, 1996;  Novak et al., 2000 |
| Aesthetic Evaluation | AE1  AE2  AE3 | I think this work is visually attractive.  I think this work has strong overall aesthetic quality.  I think the visual presentation of this work is satisfactory. | Chamberlain et al., 2018;  Leder et al., 2004 |
| Emotional Resonance | ER1  ER2  ER3 | This work elicited an emotional response from me.  I think this work has emotional appeal.  I can resonate with the atmosphere expressed by this work. | Leder et al., 2004;  Silvia, 2005 |
| Control Variables | CV1  CV2  CV3  CV4 | How would you rate your past frequency of use and experience with generative AI tools? (1 = no experience at all, 7 = very extensive experience)  How would you rate your experience with digital art or visual design? (1 = no experience at all, 7 = very extensive experience)  How interested are you in digital art creation? (1 = not interested at all, 7 = very interested)  How would you rate your general level of creativity? (1 = not creative, 7 = highly creative) | Self-developed |
| Attention Checks | AC1  AC2 | To confirm that you are reading carefully, please select the option ?somewhat agree? (6 points) for this item.  This is an attention-check item. Please directly select ?strongly disagree? (1 point). | / |

# 2. Experimental Manipulations

## Table S2. Experimental conditions and operational permissions

| Experimental design | Condition | Prompt input/revision permission | Style selection permission | Local/final editing | Maximum generation limit | Number of candidate images |
| --- | --- | --- | --- | --- | --- | --- |
| Experiment 1 | Human-only creation | Allowed (textual conception only) | Not allowed | Allowed (text box) | 0 times (AI not involved) | 0 images |
| Experiment 1 | AI-only generation | Not allowed | Not allowed | Not allowed | 1 time (completed by the system backend) | 4 images (participants selected 1 image) |
| Experiment 1 | Human-AI co-creation | Allowed (free input and revision) | Allowed | Allowed | No strict limit | 1 image per generation round (multiple rounds supported) |
| Experiment 2 | Low AI involvement | Allowed (free input) | Not allowed | Allowed | 1 time (system suggestion only) | 1 image |
| Experiment 2 | Moderate AI involvement | Allowed (input and revision) | Allowed | Allowed | 4 prompt revision and regeneration rounds | 1 image per round (up to 4 images in total) |
| Experiment 2 | High AI involvement | Not allowed | Not allowed | Not allowed | 1 time (completed by the system backend) | 4 images (participants selected 1 image) |

## Table S3. Prompt templates and AI image generation parameters

| Parameter | Setting/content |
| --- | --- |
| Base generative model | Stable Diffusion XL 1.0 (API calls conducted in March 2026) |
| Unified creative theme | A futuristic city coexisting with nature |
| Base prompt framework | A futuristic city coexisting with nature, digital art, highly detailed, masterpiece. |
| Unified negative prompt | low resolution, blurry, distorted, poorly drawn, out of focus. |
| Image output resolution | 1024 x 1024 pixels |
| Sampling steps | 30 |
| CFG Scale | 7.5 |
| Sampler | DPM++ 2M Karras |
| Style options | Cyberpunk, Watercolor, 3D Render, Oil Painting, Minimalist (available only to the co-creation and moderate-involvement groups) |

# 3. Experimental Interfaces and Material Examples

## Figures S1-S3. Experimental interface screenshots

*Figure S1. Screenshot of the interface for the human-only creation / low AI involvement condition.*

*Figure S2. Screenshot of the interface for the AI-only generation / high AI involvement condition.*

*Figure S3. Screenshot of the interface for the human-AI co-creation / moderate AI involvement condition.*

*Figure S4. Representative generated outputs*

# 4. Data Processing and Supplementary Statistical Information

## Table S4. Data cleaning and exclusion criteria

| Experiment batch | Initial sample size | Exclusion reason 1: incomplete response / abnormal duration (<180 s or >3 SD) | Exclusion reason 2: failed attention checks | Final valid sample size | Retention rate |
| --- | --- | --- | --- | --- | --- |
| Experiment 1 | 326 | 11 excluded | 15 excluded | 300 | 92.0% |
| Experiment 2 | 329 | 13 excluded | 16 excluded | 300 | 91.2% |

## Table S5. External artwork evaluation protocol and inter-rater reliability

| Protocol Item | Detailed Description |
| --- | --- |
| Evaluator background | Three independent evaluators with backgrounds in digital art and visual design; evaluators were blind to the experimental conditions. |
| Rating scale | A 7-point Likert scale was used (1 = very poor/not at all consistent, 7 = excellent/highly consistent). |
| Rating dimension 1: Novelty | Evaluated whether the work reflected distinctive and non-conventional visual ideas. |
| Rating dimension 2: Usefulness | Evaluated whether the elements and atmosphere of the work accurately matched the task theme "a futuristic city coexisting with nature". |
| Rating dimension 3: Aesthetic quality | Evaluated whether the color, composition, lighting, and other visual features of the work showed strong visual appeal. |
| Rating dimension 4: Expressiveness | Evaluated whether the work conveyed emotion and visual intention clearly and strongly. |
| Rating dimension 5: Overall creativity | Provided an overall evaluation of the creativity of the work by integrating the dimensions above. |
| Reliability index (ICC) | The intraclass correlation coefficient (ICC) among the three evaluators was ICC = 0.87, p < .001, indicating high inter-rater consistency. |

*Note: The main-effect mean differences, ANOVA results, confirmatory factor analysis (CFA) fit indices, and complete effect estimates for the bootstrap serial mediation analysis are reported in Tables 6-12 of the main manuscript.*
