# Supplementary material for: The impact of human-AI co-creation on perceived creativity in generative AI art: a serial mediation mechanism of psychological ownership, perceived agency, and creative flow
Source: Front Psychol. 2026 Oct 9;17:1986050. doi: 10.3389/fpsyg.2026.1986050 (PMC13690522; doi:10.3389/fpsyg.2026.1986050)
Supplement: Supplementary file 2 [file Supplementary_file_2.DOCX]

**Supplementary Material**

*The Impact of Human-AI Co-creation on Perceived Creativity in Generative AI Art*

**Supplementary Table S5. Condition-Level External Ratings of Overall Creativity**

| **Study** | **Condition** | **n** | **Overall creativity, M (SD)** | **Omnibus test** | **Post-hoc comparison** |
| --- | --- | --- | --- | --- | --- |
| Study 1 | Human-only | 100 | 4.16 (0.95) | F(2, 297) = 12.68, p < .001, partial eta squared = .079 | Co-creation > human-only, p < .01 |
| Study 1 | AI-only | 100 | 4.34 (1.02) | F(2, 297) = 12.68, p < .001, partial eta squared = .079 | Co-creation > AI-only, p < .01 |
| Study 1 | Human-AI co-creation | 100 | 4.81 (0.91) | F(2, 297) = 12.68, p < .001, partial eta squared = .079 | Co-creation > both other conditions |
| Study 2 | Low AI involvement | 100 | 4.12 (0.98) | F(2, 297) = 20.35, p < .001, partial eta squared = .121 | Moderate > low, p < .001 |
| Study 2 | Moderate AI involvement | 100 | 4.96 (0.88) | F(2, 297) = 20.35, p < .001, partial eta squared = .121 | Moderate > low and high, p < .001 |
| Study 2 | High AI involvement | 100 | 4.41 (1.03) | F(2, 297) = 20.35, p < .001, partial eta squared = .121 | Moderate > high, p < .001 |

*Note.* Overall creativity was rated on a 7-point scale by three condition-blind evaluators. Values are aggregated evaluator means. Inter-rater reliability for the external evaluation was ICC = .87, p < .001. Omnibus tests are one-way ANOVAs; post-hoc comparisons use the study-level tests reported in the manuscript.
